# Heart-Rate Variability—More than Heart Beats?

**DOI:** 10.3389/fpubh.2017.00240

**Published:** 2017-09-11

**Authors:** Gernot Ernst

**Affiliations:** ^1^Anaesthesiology, Pain and Palliative Care Section, Kongsberg Hospital, Vestre Viken Hospital Trust, Kongsberg, Norway

**Keywords:** heart-rate variability, autonomous nerve system, sympathetic nerve system, parasympathetic nerve system, systems theory, time series

## Abstract

Heart-rate variability (HRV) is frequently introduced as mirroring imbalances within the autonomous nerve system. Many investigations are based on the paradigm that increased sympathetic tone is associated with decreased parasympathetic tone and *vice versa*. But HRV is probably more than an indicator for probable disturbances in the autonomous system. Some perturbations trigger not reciprocal, but parallel changes of vagal and sympathetic nerve activity. HRV has also been considered as a surrogate parameter of the complex interaction between brain and cardiovascular system. Systems biology is an inter-disciplinary field of study focusing on complex interactions within biological systems like the cardiovascular system, with the help of computational models and time series analysis, beyond others. Time series are considered surrogates of the particular system, reflecting robustness or fragility. Increased variability is usually seen as associated with a good health condition, whereas lowered variability might signify pathological changes. This might explain why lower HRV parameters were related to decreased life expectancy in several studies. Newer integrating theories have been proposed. According to them, HRV reflects as much the state of the heart as the state of the brain. The polyvagal theory suggests that the physiological state dictates the range of behavior and psychological experience. Stressful events perpetuate the rhythms of autonomic states, and subsequently, behaviors. Reduced variability will according to this theory not only be a surrogate but represent a fundamental homeostasis mechanism in a pathological state. The neurovisceral integration model proposes that cardiac vagal tone, described in HRV beyond others as HF-index, can mirror the functional balance of the neural networks implicated in emotion–cognition interactions. Both recent models represent a more holistic approach to understanding the significance of HRV.

## Introduction

The human body consists of many different interacting systems. The brain would not survive without circulation, and circulation is only possible when energy metabolism is working. Eating food is only safe when the liver extracts the toxic substances and the immune system defends against pathological germs, and so on. In reductionist bioscience, the human physiology is investigated by identifying its parts and focusing on one of it. In controlled experiments, one factor is changed and every other possible controlled as much as possible. This approach has been extraordinarily successful in understanding basic principles of human (or animal) physiology. In principle, the reductionist approach assumes that if scientists investigate every part of the human body and its possible interactions with other parts and when they put all together they will understand human physiology.

This approach does not necessarily work out. Even reductionists agree that a brain will die within 3 min when circulation breaks down. However, the reductionist approach does not appreciate the tight interconnection of different parts of the body to function in real time. The basic physiological approach based on research in the nineteenth century is still valid when looking at some general functions. Heart function can be described by a linear equation where cardiac output is multiplied with heart frequency. The view changes, however, when the level of detail is increased. Every heart beat is at least slightly different; cardiac output is influenced by factors as simple as preload, afterload, or systemic resistance, or as complicated as a plethora of humoral factors, a variety of efferent signals and the condition of the heart muscle. Neural inputs are never the same; their patterns are depending on afferent signals from the heart to different brain centers and again humoral signals. These are modified by energy metabolism, immunological signal cascades, the internal state of the brain itself. It is possible to identify hundreds of different factors which probably influence the cardiac output in various ways. However, it is also possible to turn it around. The cardiac cycle has effects on brain function beyond simply providing enough blood flow. Oscillations in the brain are coupled to the heartbeat and oscillations in other systems. The state of the immune system has effects on the brain, which again has effects on the circulation system, apparent in sepsis, but relevant also in less dramatic situations. It turns out that the human body consists of closely interconnected parts which communicate closely in real time on the level of changes in time orders between milliseconds and hours, even days.

Another part of the picture regards the influence of perturbations. In a linear, reductionist world, a change in one condition will have a straightforward effect on the connected system. Increase adrenaline twice, and the heartbeat increases twice. Take away some blood, and the blood pressure decreases, take away twice as much, and the blood pressure decreases twice as much. The reality is different. A change of internal or external factor will often only induce minor changes. Losing a half liter of blood will probably induce small changes in blood pressure and slightly increase heart frequency. Removing more will eventually lead to sudden changes, at the end to a breakdown of circulation.

Meet another paradigm. The human body can be regarded as a system. A system can be defined as a set of interacting or independent components forming a whole. Each system has boundaries defining an inside and an outside. Systems again can consist of a set of systems (subsystems) interacting in the same way. This definition can be applied to physical entities, human bodies, or social constructions in the same way. Systems can be studied by breaking them up into parts as reductionists do, but the notion of the system includes that a system “is more than its parts” (1). Systems, in general, have some properties in common. They can be remarkably stable against perturbations, but they also can be remarkable fragile in case of a specific perturbation, leading to a sudden change. This possible change is frequently termed phase change, and the ability to change is termed emergence. Apparently, sudden system changes due to minor changes in perturbations are not linear, but non-linear. This means simply that a small perturbation might cause a major system change, or a significant perturbation only a minor modification. The advantage using this approach to the human body is that both a sophisticated understanding and sophisticated tools to investigate systems have been elaborated (2, 3).

Biomedical research in the last decades has reached an enormous level of detail. Independent of the area of investigation, the knowledge of genetics, translational mechanisms, intracellular pathways, receptor systems and their ligands, or immunological mechanisms have reached a level where it is challenging to understand the system as a whole. It turned out that simple systems are not as simple, physiological systems are best assumed to be more complex than at first apparent until it can be demonstrated otherwise (2). All physiological systems can be understood in a cybernetic way. They consist of control cycles using either positive or negative feedback mechanisms which were identified in the second half of the last century. Systems with more than three positive and negative feedback circles in combinations are often not predictable; they show complex behavior.

At the end of the last century, the increasing level of details provided by biomedical research provoked debates how to understand the whole system. Similar debates also occurred in other scientific disciplines. System theory and cybernetics gradually evolved to something which was termed complexity science although an exact definition was challenging and no unified theory existed. At the same time period, system theory evolved to dynamic system theory beyond other influences also with the help of inputs from chaos theory which in reality investigates the behavior of deterministic non-linear systems (3). Another evolving direction of investigations was based on increasing computational power which it made possible to simulate large artificial systems in real time. Agent base modeling, cellular automata, and genetic algorithms were developed. All these approaches made contributions to understand complex systems; main tools repeatedly used were an analysis of time series of signals and computer simulations (4).

In the beginnings of 2000s systems biology was established. It is a biology-based inter-disciplinary field of study focusing on complex interactions within biological systems on several levels—intracellular, intercellular, hormonal, macroscopic—using a comprehensive approach and as tools data mining, mathematical models, and time series analysis (4, 5). A time series is a sequence of observations made over time, in the case of heart-rate variability (HRV) heartbeats, but also other parameters (like blood sample results, EEG signals, etc.) (6). Time series analysis can help to identify hidden patterns and even causalities in physiological systems (7). Systems biology analyses different parts of the body as a kind of network (8). Most investigations are conducted on the subcellular level, looking on metabolic and genetic regulation networks and then analyzing them with the help of sophisticated network methods, which again are validated with experimental data. Network analysis or models can even be applicated in the case of unknown quantitative relationships or lacking precise data (e.g., By using Boolean networks) (9, 10).

This review is organized as follows. After a short introduction of HRV, some physiological systems with influence on HRV will be reviewed. In the next part, different paradigms of HRV are presented—HRV as time series analysis, as a proxy for the autonomic vegetative systems and as a proxy for a complex regulatory system. Finally, implications will be discussed.

## Principles and Parameters of HRV

The principles of HRV are simple. The heartbeat is measured, usually with the help of an ECG signal which is obtained with an adequate device. A minimum sampling rate between at least 250 and 500 Hz is recommended (11). QRS-distances are measured (called NN-distances) after identification of ventricular and supraventricular extrasystoles which usually are interpolated. Subjects with a high rate of extrasystoles or atrial fibrillation (AF) are usually not feasible for analysis [but see Ref. (12)]. At the end of the measurement period, a time series of milliseconds can be processed in time domain, frequency domain, geometrical measures, and different non-linear measures like fractal parameters or different calculations of entropy (13). The most common used parameters are the SD of NN Intervals (SDNN) and root means successive square difference (rMSSD), calculated by squaring of each NN interval, thus calculating the mean value and drawing the square root (14). Geometric methods are derived from sequences of NN intervals. Different geometrical methods include the 24-h histogram, the HRV triangular index, the triangular interpolation of NN interval histograms, and methods like the Poincaré-plot. Frequency domain (power spectral density) describes the periodic oscillations of the heart-rate signal, transformed into different frequencies areas, and returns numerical values about their relative intensity (14). Most frequently, the frequency-domain parameters are calculated non-parametrically with the fast Fourier transformation. The most used parameters are Total Power, VLF (very low frequency, <0.003–0.04 Hz), LF (low-frequency power, 0.04–0.15 Hz), HF (high-frequency power, 0.15–0.4 Hz), and the ratio LF/HF. HF is often understood as a proxy for the parasympathetic nervous system (PNS). It can be influenced by the frequency of breathing or pathological forms of respiration, but is reliable with normal respiration (15) and is to a certain grade similar to respiratory sinus arrhythmia (RSA) (16). Both the sympathetic nervous system (SNS) and the PNS modulate LF. High LF reflects often increased sympathetic activity. The LF/HF ratio might reflect the global sympathetic/vagal balance. VLF is probably influenced by the renin–angiotensin system and is also associated with the sympathetic activity (15, 17). Non-linear methods often focus on (self)similarities in the time series. Classical algorithms for the analysis of self-similarities are fractal methods. Different forms of entropy measures have been used (18). A standard for measurement and interpretation was published in 1996, and most studies afterward are based on it (11).

## HRV is More than the Autonomic Nerve System: Some Physiological Systems with Influence on HRV

### Autonomic Nerve System

The autonomic nervous system (ANS) is an important part in the control of different physiological systems, e.g., the heart, smooth muscles, endocrine, and exocrine glands. It has an afferent (sensory) and efferent parts and is distinct from the somatic nervous system in several ways. The main function of ANS is homeostasis, largely regulated by autonomic reflexes, (almost) not under voluntary control. Sensory information is frequently transmitted through afferent vegetative nerve fibers to homeostatic control centers, processed and specific reactions are sent through efferent vegetative fibers. The ANS has as mentioned specific transmitter substances—mostly acetylcholine (ACh) and norepinephrine (NE)—corresponding receptors and can be divided into preganglionic and postganglionic fibers. The central control of the vegetative nerve system has been identified in several subdivisions of the hypothalamus, but several other brain regions including the association areas of the limbic cortex, the amygdala, and the prefrontal cortex are also connected to these hypothalamus nuclei.

The hypothalamus itself controls two more systems in addition to the ANS, the endocrine system and an ill-defined neural system involved in motivation (19) and social behavior ((20, 21)). The ANS has three major divisions: sympathetic (SNS), parasympathetic (PNS), and enteral (the latter is often underestimated). In a traditional view, the sympathetic and the parasympathetic systems are opposed to each other. In this view, the SNS is responsible for stress reactions and the PNS for relaxing. All visceral reflexes are processed by local circuits in the spinal cord and brainstem (22). The sympathetic system’s phasic activity is triggered by (positive and negative) stress and increases cardiac energy demand by increasing heart frequency and contractility through binding of NE to adrenoreceptors on cardiomyocytes (23). The parasympathetic system’s more tonic activity maintains homeostatic heart frequencies and contractility without exhausting, triggered by the release of ACh binding directly to muscarinic receptors on cardiomyocytes and also on nicotinic receptors on postsynaptic neurons (24, 25). The PNS reacts faster on external and internal changes, within 1 s, whereas the SNS reacts after >5 s (26). The role of the ANS in the regulation of heart function is important, but much more influences exist, which makes it to a complex system with several likewise complex subsystems. The following interactions with other systems are only examples.

### Sinoatrial Node

The sinoatrial node is, of course, the origin for the pace of the heart. It can, however, itself be considered as a system of weakly coupled oscillators with self-organizing properties, synchronized by a mechanism of mutual entrainment or phase locking (27).

Already on the intracellular level, cell organelles behave as weakly coupled oscillators. A combined experimental and simulation study showed with the help of two photon laser scanning microscope an oscillating network behavior of cardiac mitochondria, distinctly different from random behavior in the form of an inverse power law typical for fractal behavior. They might play a role as an intracellular timekeeper and have a long-time memory function of the oscillations, suggested by a calculated fractal dimension close to 1.0 (28). This kind of network behavior is of particular importance when HRV is interpreted within a complexity theory paradigm (8), as discussed below.

Cardiac neurons are localized both in the heart as intrinsic neurons and intrathoracically. They form a local distributive network, controlled by brainstem and spinal cord neurons and processing both central and local information to control the heart (29). Major intrinsic cardiac ganglionated plexus have sensory neurons responding to metabolic changes within particular heart regions (30). Such sensory inputs might be responsible for the generally stochastic behavior displayed by many atrial and ventricular neurons (31). In the same way as intracellular organelles, intrathoracic neurons have long-time memory properties based on cardiovascular events during the last subsequent cardiac cycles and influence efferent neuronal inputs (29). Because of this, perturbations can have effects over the next few cardiac cycles already based on the coupling of intrathoracic neurons. Because of multiple feedback circles complex behavior is already existent on this level. Typical for a complex system, its behavior is robust even when some subpopulations are compromised (32, 33).

### Respiratory System

One of the leading causes of sinus arrhythmia is probably central coupling of respiratory drive to cardiac vagal motor neurons (34–36). Medullary respiratory neurons provide efferent signals to medullary sympathetic premotor neurons (36).

The term RSA is used to describe the fluctuation of heart rate during the respiratory cycle. It is highly dependent on the vagal tone in the heart and is observable at a frequency band of 0.15–0.4 Hz. Usually, RSA is interpreted to mirror the vagal activity (37), involving several interaction levels. Beyond others, the fluctuations of blood pressure due to changes in intrathoracic pressure during the respiratory cycle have been discussed as one of the most important mechanisms of RSA (38). The baroreflex—a rapid feedback loop where elevated blood pressure results in decreased heart rate and decreased blood pressure decreases baroreflex activation—has been associated with RSA, but some evidence indicates that the baroreflex is mostly involved in upright, but not in supine position (where HRV frequently is obtained) (37, 39, 40). An alternative explanation is based on the notion that neural networks generating the respiratory drive have also influence on oscillatory patterns in the vagal and sympathetic outflows, as already proposed several decades ago (41–43).

A classical interaction between the respiratory and cardiac system occurs in congestive heart failure, present in more than 50% of the patients (44). The pathophysiology of Cheyne–Stokes respiration is based on the combination of low cardiac output, pulmonary congestion, and high sympathetic activation. Both congested lungs and sympathetic hyperactivity lead to hyperventilation causing a decrease in arterial CO_2_ to a level below the apneic threshold. The hyperventilation pattern consecutively becomes periodic because the diminished arterial CO_2_ reaches the brainstem delayed due to the low cardiac output. When first the low partial pressure of CO_2_ is detected, respiration drive is stopped until CO_2_ increases. This again is detected late, which results in hyperventilation until CO_2_ is again on a low level and a new cycle begins (45). The increased sympathetic drive is in particular caused by increased CO_2_ partial pressure in the blood (46). The significance of Cheyne–Stokes respiration might be a mechanism to improve the efficacy of pulmonary gas exchange by phase-locking heart beats with phasic hyperpnea within the respiration cycle length (47). Cheyne–Stokes respiration again affects both sinus rhythm and AF. The latter does usually not react to normal ventilation, possibly due to changes of the atrioventricular nodal refractory period (48, 49).

### Endocrinological System

In difference to other pathological conditions, endocrinological diseases can be associated with increased HRV parameters. Subjects with increased sodium excretion associated with an increased number of CYP11B2-344T alleles showed a higher LF/HF ratio, but not subjects with the AT1R 1166C allele. Increased sodium excretion correlates with expanded plasma volume which might explain the effect on the parasympathetic tone (50). Cortisol concentration is negatively correlated with HRV (51). Estrogen increases the parasympathetic parameters and progesterone sympathetic parameters of HRV (52, 53). Oxytocin application increase (rather moderate) HF and detrended fluctuation scaling exponent (54).

### Immunological System

Infection, injury, or trauma causes an inflammatory reaction in the body which aims to restore homeostasis. The inflammatory response of the host is based on a complex combination of different immune mechanisms contributing to the neutralization of the invading pathogens, the restoration of injured tissues and to wound healing (55). The first steps of inflammatory reactions involve the release of pro-inflammatory mediators, especially, interleukin (IL)-1 and tumor necrosis factor (TNF), but also adhesion molecules, vasoactive mediators, and reactive oxygen species. This first release of pro-inflammatory cytokines is initiated by activated macrophages and is considered as crucial to trigger local inflammatory response (56).

Excessive production of cytokines, such as TNF, IL-1, and high mobility group B1, however, is causing more damage than invading pathogenes, like in the case of sepsis where immune reactions cause tissue injury, hypotension, diffuse coagulation, and in a high proportion of patients, death (57). Therefore, the inflammatory response needs to be balanced which is based on the nearly simultaneous release of anti-inflammatory factors like the cytokines IL-10 and IL-4, soluble TNF receptors, and transforming growth factor (TGF-beta). Using the terms pro- and anti-inflammatory is, however, rather simplistic, but widely used in the discussion of the complex cytokine network. If the local inflammation increases, TNF, and IL-1 β starts to circulate in the blood and other body fluids. This has major consequences for the CNS because these molecules are also signal molecules for the activation of brain-derived neuroendocrine immunomodulatory responses. Another superordinate control instance of the immune reaction is based on neuroendocrine pathways, as the well-known hypothalamic–pituitary–adrenal axis, but, usually underestimated, the sympathetic division of the ANS (SNS) (58, 59). The CNS is also able to control inflammation and contributes to the other anti-inflammatory balancing mechanisms (55).

The cross talk between the immune system and the brain relies, therefore, not only on classical humoral pathways but also substantially on recently discovered neural pathways. Vagus nerve afferent sensory fibers play a vital role in the communication between body and brain when inflammation is occurring. Immunogenic stimuli stimulate vagal afferents both directly by cytokines released from dendritic cells, macrophages, and other vagal-associated immune cells, and indirectly through the chemoreceptive vagal ganglion cells (55).

Acetylcholine plays a major role as neurotransmitter and neuromodulator in the CNS. ACh is an important transmitter substance in ganglion synapses of sympathetic and parasympathetic neurons and is the main neurotransmitter in the postganglionic parasympathetic efferent neurons. Two types of receptors have a high affinity to ACh: muscarinic (metabotropic) and nicotinic (ionotropic). However, like other mediator substances as opioids, ACh is also involved in immune responses. RNA for muscarinic and nicotinic receptors has been detected in mixed populations of lymphocytes and other cytokine-producing cells (60, 61).

A majority of the cells are also capable of producing ACh (62). ACh has an anti-inflammatory effect, beyond others because ACh decreases TNF production *via* a posttranscriptional mechanism. ACh blocks also the release of other endotoxin-inducible pro-inflammatory cytokines, such as IL-1b, IL-6, and IL-18, by same mechanisms; it does, however, not suppress the anti-inflammatory cytokine IL-10 (63, 64). In several experimental models of sepsis, myocardial ischemia and pancreatitis, all characterized by an excessive immune reaction, vagus stimulation was sufficient to block cytokine activity (65–67). The vegetative system may, therefore, play a major role in the immune defense (68). This works in both ways: changed activity of the vagal system modulates the inflammatory response by increasing the release of transmitter substances in the synaptic space like noradrenaline or ACh. On the other hand, inflammatory influences can also enhance or block vagal activity. Pro-inflammatory cytokines activate vagal afferent signaling which again activates efferent vagal signaling directly through the nucleus of the solitary tract (NTS) or indirect through NTS neurons activation of vagal efferents in the dorsal motor nucleus. The vagal system can be considered as an inflammatory control circuit for the inflammatory status in the periphery (69). If this system in animals is destroyed, they are more sensitive to endotoxemic shock (55). The area postrema, a region in the brain stimulated by increased blood concentrations of IL-1 beta can also activate the cholinergic anti-inflammatory pathway (70).

Sepsis is a life-threatening condition, usually caused by invasive bacteria. Success in treatment depends on early identification and treatment with appropriate antibiotics (71). Sepsis is traditionally diagnosed with the help of the clinical picture and blood samples of immunologic parameters (72). HRV changes are sometimes the earliest measurements before the first clinical effects of sepsis are observed (73, 74). This might be based on the close interaction between the PNS and the immune system, as described. HRV parameters change under inflammatory conditions. Soluble TNF-α receptors and IL-6 correlate (negatively) with time-domain HRV variables (SDNN, SDANN) (75–77), also endothelin 1 blood concentration is negative correlated with TP and ULF (78). Although TNF-α might not be associated with HRV variables, a clear relation between IL-6 and decreased HRV has been demonstrated (79). The liver releases CRP as a response to increased IL-1 and Il-6 concentrations, decreased HRV parameters are associated with increased CRP (80–83). In both newly diagnosed and chronic diabetic patients, increased IL-6 is correlated with decreased time domain (SDNN) and frequency-domain parameters (84). In a long-time cohort study with a follow-up of 15 years, linear HRV parameters and DFA was associated with inflammatory parameters at baseline. VLF, LF, TP, and SDNN was negatively correlated with CRP, Il-6, and WBC, DFA had and an inverse association with Il-6 and CRP, and HRT slope to WBC and Il-6 (85).

In conclusion, inflammatory parameters, such as IL-6, CRP, and TNF-alpha, correlate negatively with different HRV parameters. This is not only observed in classical “parasympathetic” parameters like rMSSD or HF but also for more general or “sympathetic” parameters like SDNN, SDANN, TP, VLF, and LF (86). The immune system is a still underestimated physiological and pathophysiological cause of HRV dynamics.

### Metabolic Function

Insulin is a major player in metabolic function. In the heart, two different insulin signaling pathways have been identified: the phosphatidylinositol-3-OH kinase pathway, predominant in metabolic tissues and the growth-factor-like pathway (mediated by mitogen-activated protein kinase). Insulin resistance in the heart inhibits the metabolic pathway and stimulates the growth factor-like pathway (87, 88). This leads to decreased glucose uptake with possible consequences for cardiac cell metabolism (88, 89) and is a rather complex process also involving coagulation factors and the immune system (88). Already isolated obesity is associated with increased release of cytokines and other inflammatory markers like the intercellular adhesion molecule-1 (90). The islets with insulin-producing beta cells in the pancreas are innervated by both sympathetic and parasympathetic neurons, making possible a direct control by the CNS. This might also indicate that central nervous circuits have a major role in the functional adaptation to changes in insulin sensitivity. When the ventromedial hypothalamus is lesioned in experiments, increased vagal activity is observed and insulin is released, which can be blocked by vagotomy (91). The PNS effect on the beta cells is mediated by ACh and its effect on the M2 muscarinic receptor. Activation of the SNS through the α2-adrenergic receptor is associated with decreased insulin release, stimulation of β-adrenergic receptors enhance insulin output (92, 93).

There are several factors which can affect HRV in acute and chronic metabolic changes. The direct involvement of the vegetative nerve system can be attenuated in more chronic conditions, when the diabetic autonomous neuropathy, which necessarily occurs after a certain length of illness (94). HRV is an established tool in the diagnoses of diabetic neuropathy. During its sub-clinical phase, HRV can help in detecting cardiac autonomic neuropathy before the disease is symptomatic (95). Interestingly, reduced HRV in diabetes might be related to increased glycemic variability (96). Theoretically, this could be explained with the help of the concept of coupled oscillators—when the HRV-system as one oscillator fluctuates less, the coupling decreases which again allow the glycemic system to fluctuate independently with less control.

The LF parameter of HRV has been used to predict hypoglycemia (97), this might be even work in patients with advanced diabetic neuropathy (98). Parameters like SDNN and rMSSD are reduced when glucose and insulin are elevated (99). Regarding lipid metabolism, change of diet has been associated with HRV changes (100), but reports are conflicting regarding possible correlations between lipid concentrations in blood and HRV (101, 102).

## HRV as Time Series

Time series show the variation of a parameter during a period. In complexity theory, they are considered as surrogates of the particular system, giving information about the state of the system. Typical time series can consist of a series of blood samples (e.g., CRP, creatinine, white blood cell counts), physical measurements like heartbeats, blood pressure, EEG; or movements like gait, eye blinking or variability of breathing patterns. Even short time series can also give information about a system. Usually, most properties of time series are ignored. They are summarized by simple statistical calculations like the mean and the SD. In the case of HRV, several linear and non-linear algorithms were eventually introduced. The research literature can be roughly divided into three areas. In the first, HRV parameters are used as a prognostic tool. Short-term or long-term results in trials are associated with (usually decreased) HRV parameters. This approach has been very successful providing studies where an analysis of only 2 min of heartbeat predicted all-cause mortality many years later, e.g., in Ref. (103–105). The second approach uses HRV as a proxy for the state of the vegetative system, based on the century-old notion of a balanced situation of the sympathetic and the parasympathetic part. The third approach relies on the complexity paradigm. It considers variability as a proxy for the health of a system and decreased variability as a sign of a pathological condition. In the next section, I will discuss the scientific evidence for associations between HRV and the state of the vegetative system, followed by a section where I present the third approach.

## HRV as Proxy for the Vegetative Nerve System

Heart-rate variability is very often used to describe the activity of the SNS and PNS. This is based on the assumptions that there is something like a general activity level of the ANS, that HRV parameters mirror this activity and that SNS and PNS are in the balance, meaning that high SNS activity is associated with low PNS activity and *vice versa*. If this is the case, it should be possible to measure consistent patterns in the activity of different vegetative efferents. But this supposed consistency has frequently not been found. Different other parts of the SNS have not necessarily the same kind of oscillations as the cardiac part of SNS if they have oscillations at all. Cutaneous vasoconstrictor fibers, sudomotor fibers, adrenaline-regulating adrenal preganglionic neurons, and nerves supplying the brown adipose have not oscillations as observed in other parts of the SNS (106–109).

The sympathetic output might also be as similar in different parallel measured subsystems (110). The physiologist William Cannon propose at the beginning of the last century a model of autonomic control, where either sympathetic activation is high and parasympathetic activation is low or *vice versa* (111). In contradiction to this frequently used model evidence has been provided that descending influences from higher neural systems can trigger all patterns of changes in the SNS and PNS, whether reciprocal, independent or even co-active (112, 113). Already early observations indicated that both divisions of the vegetative nerve system could be similar active and work together with the somatic motor system to regulate most behavior, both in normal and in emergency conditions (114). Even when either the SNS or PNS are predominant in the control of an organ or anatomical region (110) and even when the sympathetic and the parasympathetic division have opposed effects on these areas, the balance is more complicated. Coactivation or a degree of opposing activations patterns can both be important to keep homeostasis when external conditions change (22). A major challenge is to interpret experimental approaches which often use isolated organ systems in rather artificial experiments, where other systems are lacking.

The classical description of a stress response of the SNS includes increased heart beat frequency, vasoconstriction, piloerection, and pupillary dilatation (115). However, stress response can be independent of SNS activation.

The increased muscle blood flow, for instance, is triggered by activation of a cholinergic vasodilatation pathway, with only minor involvement of adrenergic pathways (115, 116). Also during the diving reflex, simultaneous activation of the sympathetic and parasympathetic part of the ANS has been observed. When the head is under water, it causes a massive activation of trigeminal afferents nerve fibers which again initiates an intense and simultaneous reaction of SNS and PNS. The SNS triggers massive vascular constriction everywhere (except brain and heart), the vagal activation triggers severe bradycardia and decreases cardiac contractility. The meaning of the diving reflex is to reduce energy demand in a stress situation with an anticipated lack of oxygen in the body (117, 118). Interestingly, the intense simultaneous activation of SNS and PNS is associated with a dramatic loss of fractal properties of the HRV signal (119). Another example is the simultaneous pathological influence of SNS and PNS on AF (120), probably underestimated because HRV usually cannot be used as a tool in these patients. A daily observation in the operation theater is the change of heart rate caused by a sudden nociceptive input in a patient with superficial anesthesia: skilled anesthetists notice a sudden moderate decline of heart rate following by a marked increase some seconds later, most probably caused by simultaneous activity increase of the SNS and PNS, most marked observed in strabism surgery (121). Hundred years after Walter Cannon’s insights, we should accept that high SNS activity does not necessarily imply a low PNS activity.

Another important question is if the different parameters of HRV mirror the state of the ANS. It has been shown, that representations of ANS in the brain and brain centers involved in HRV are mainly the same (122–125). However, does HF correlate with the parasympathetic tone? The presented evidence often consists of pharmacologic studies where the sympathetic system is blocked with, e.g., atropine (126–128). In a complex experiment, the relationship between HRV parasympathetic activity was described by a function with an ascending part that goes over to a plateau level (129). These results have been challenged, and some authors argue that HF is a possible, but not optimal index (130), or that it only works out when respiration is controlled (131). Similar in the case of LF and sympathetic activity: several studies confirm the relationship (128, 132, 133), but not in all subjects (134) and some results were challenged (135). Contradictory results are, e.g., when an intense training session in healthy persons (which should usually increase sympathetic activity) is associated with a decrease of HRV parameters (136). High dose atropine is expected to block completely vagal parasympathetic activity and according to Cannon’s model increase sympathetic activity, but it nearly all LF power (and HF power) disappeared in an investigation (126, 127). Most of the studies regarding HRV components and different parts of ANS were conducted more than 20 years ago. Since then, the debate has calmed down, and these associations have been assumed by most authors using HRV. The relation between HRV parameters and ANS does certainly exist, but the reality is probably more complicated and more research is needed.

## HRV as System Indicator

Kauffman, affiliated with the renowned Santa Fe Institute for Complexity Research, proposed the notion of life being generally at the edge of chaos (137, 138). Based on theories of complex (adaptive) systems, life is defined being at the frontier between mathematical chaotic behavior and (some kind of) order. Order is here defined as the possibility to estimate the behavior of the system based on its internal states during the last period. The same argumentation is used of some authors arguing theoretically that “health” is defined not being in complete thermodynamic equilibrium; too close proximity (decreased variation, too little energy dissipation, low entropy) or too far distance (increased variation and energy dissipation, high entropy) would indicate pathology (139). Decreased HRV parameters are usually considered as pathological, according to a notion of Goldberger (140). Changed HRV variability, as seen in elderly individuals is explained as reduced number of system components and reduced coupling between elements in a complexity theories paradigm (141, 142). Many studies seem to support this paradigm, but there are also some contradictory reports where increased variability is associated with illness. In endocrinological diseases, HRV can be increased compared to healthy controls. In patients with acromegaly (caused by increased levels of growth hormone due to hypophysis adenoma), the amount of growth hormone release over 24 h correlates with higher approximate complexity (143). Also in patients with Cushing syndrome, approximate entropy is greater when ACTH and cortisol concentration levels are increased (144). Using the same algorithm on a time series of hormone concentration alone has also shown higher approximate entropy of luteinizing hormone and testosterone concentrations in older males (145). Several diseases have been associated with decreased or increased complexity, compared to healthy subjects (142).

Porges introduced a model with an evolutionary approach (146–149). According to it, the autonomous system has three neural circuits: the myelinated vagus, the unmyelinated vagus, and the sympathetic–adrenal system (Table 1). All three circuits are involved in the regulation of physiological states. The sympathetic–adrenal part is mainly involved in mobilization related behaviors (fight and flight) whereas the vagal circuits are related to immobilization related behaviors. This approach can be discovered already in Cannon’s classical description (111), but Porges extended it to social behaviors. Initially, a system mainly aimed at homeostasis, ANS became during evolution increasingly important in the regulation of social behavior, according to this theory. The polyvagal theory proposes thus that the development of the mammalian ANS provides the neurophysiological substrates for the emotional experiences and affective processes that are major components of social behavior (147).

**Table 1:** Three phylogenetic stages of the neural control of the heart, as proposed of the Polyvagal Theory; modified from Ref. (147).

Phylogenetic state	Autonomic nervous system component	Behavioral function	Lower motor neurons
I	Unmyelinated vagus	Immobilization, passive avoidance	Dorsal motor nucleus of the vagus
II	Sympathetic–adrenal	Mobilization (active avoidance)	Spinal chord
III	Myelinated vagus	Social communication, self-soothing and calming, inhibit sympathetic–adrenal influences	Nucleus ambiguus

Thayer’s approach is based on the already mentioned complexity theories paradigm. He proposes to use HRV as a measure of the degree to which a system provides flexible, adaptive regulation of its component systems, with other words as a measure of the adaptivity of the brain–body system (150, 151). They describe their view as follows: “When processes mutually constrain one another, the system as a whole tends to oscillate spontaneously within a range of states. The various processes are balanced in their control of the entire system, and thus the system can respond flexibly to a range of inputs. However, such systems can also become unbalanced, and a particular process can come to dominate the system’s behavior, rendering it unresponsive to the normal range of inputs (…) A system, which is ‘locked in’ to a particular pattern, is dysregulated” (124). This approach has been developed further to a model called Neurovisceral Integration Model (124, 152, 153). Thayer and Lane proposed at the beginning that HRV might be a biomarker for emotion regulation, but they extended it eventually as a surrogate parameter for the more general top-down ability to self-regulation which is again coupled to the vagus nerve to the heart. As a general central nervous base for self-regulation, a Central Autonomic Network (CAN) has been defined (154). This network consists of parts of the prefrontal cortex (anterior cingulate, insula, orbitofrontal, and ventromedial cortex), limbic structure (amygdala and hypothalamus), and brain stem (periaqueductal gray matter, nucleus ambiguus, ventrolateral, and ventromedial medulla). These coupled structures and its oscillatory signal patterns are integrated into the nucleus of the solitary tract (NTS) and again coupled through efferent parts of the vagus nerve with organs outside the brain. The coupling is bidirectional such as peripheral oscillations in the heart, lung, but also in the immunological system (and others) can lead to changes in the CAN. According to the model, this regards especially the parasympathetic activity and should be associated with variations of the HF signal. This model has recently been expanded by defining eight levels of vagal control beginning with intra-cardiac control on the lowest level up to the highest levels where interactions between different parts of the prefrontal cortex shape the vagal tone over longer time periods (152). The model is sophisticated and based on new neuroscientific evidence. Especially on the network level, research is still needed. A recent investigation, for instance, reported no association between different individual levels of HF and general activity in the default mode network or the salience network in the human brain (155).

Besides these two models, other theories have been published. Grossman’s biological behavioral model focuses on the regulation of energy exchange by synchronizing respiratory and cardiovascular processes during metabolic and behavioral changes. Also there the main component is vagal signals, reflecting functional energy reserves as adaptive capacity (156). Lehrer and Gevirtz’s model is interested in the effects on slow pace breathing to increase vagal tone as a beneficent surrogate for health, mainly as a possible explanation for the effects of HRV biofeedback (157). McGraty and Childre have published a similar model within a broader context of physical and mental health (158).

Based on both approaches several studies have been published, mainly focusing on emotion regulation and executive control. Newer approaches also include sociodemographic co-variables (159), indicating that lower vagal components HRV mirror reduced adaptivity in self-regulation, supposed to be associated with lower sociodemographic status. This would indicate that HRV could be applicated as proxy for executive control and might be even used as measurement for therapeutical inventions. An association of lower sociodemocratic status and lower executive function, however, has been questioned in recent work (160).

An underreported issue is consequences of relatively higher HRV indices, based on the interest for the association between lower HRV and pathological conditions. Some evidence for that relatively higher HRV is associated with better mental and physical health has been reported. For instance, higher rMSSD is associated with better self-rated health (161).

## Implications for Clinical Praxis and Further Research

A main issue in HRV research is that most studies report HRV parameters, but the main research question was not related to HRV. Frequently, relevant information or non-significant results are not reported. Relevant co-variables beyond age and gender, like Body Mass Index, physical status, or social background are often omitted, at least in the control groups. The well-documented association between immune status and different HRV results, for instance, is rarely reported, e.g., in the form of CRP values.

In the case of time series analysis, linear parameters have been established and are being used reliably. The non-linear part, however, is more difficult. A wide number of different forms of fractal measures, entropy measures or other non-linear indices have been introduced in the last two decades, but their relevance is still unclear. The relation between different HRV indices and the activity of various sympathetic and parasympathetic subsystems is also still unclear. In the last years, HRV parameters (especially HF) mirroring the function of the executive brain, in particular in the prefrontal cortex has been investigated. As pointed out by Holzman and Bridgett (162), a small, but a significant relation between top-down functions of the prefrontal cortex and HRV is now established, but more exact definitions and its clinical relevance remain to be shown.

Heart-rate variability is a fascinating observation and insight in its mechanisms is increasing. One main issue remains that interindividual differences are high and statistical effects are usually shown only on the group level. In particular regarding psychologic functioning, although an association between lower HRV and lower adaptivity is now established (162), the differences are frequently, although significant, very tiny—see as example recent work on HRV and depression (163), which makes its clinical application difficult.

From a systems science perspective, HRV measurement begins to be a sophisticated and relevant tool for both scientific and clinical insights.

## Author Contributions

GE has elaborated and written the review.

## Conflict of Interest Statement

The author declares that the research was conducted in the absence of any commercial or financial relationships that could be construed as a potential conflict of interest.
